# A Frequency-Aware Dual-Stream Deep Learning Framework for Athlete Workload Monitoring and Injury Risk Assessment: A Multi-Dataset Validation Study in Professional Team Sports

**DOI:** 10.3390/s26134228

**Published:** 2026-07-03

**Authors:** Jinnian Tong, Peng Gao

**Affiliations:** 1 Graduate School, Dongshin University, Naju 58245, Republic of Korea; jinniantong@dsu.ac.kr; 2 Department of Physical Education, Shandong Vocational University of Foreign Affairs, Weihai 264504, China

**Keywords:** frequency-aware modeling, injury risk prediction, athlete workload monitoring, bidirectional LSTM, spectral decomposition, cross-modal attention fusion, sports digital engineering, multi-dataset validation

## Abstract

The accumulation of training and competition loads represents a critical determinant of musculoskeletal injury risk in professional team sports, yet contemporary monitoring systems remain limited by their reliance on single-domain temporal analysis that overlooks the multi-scale rhythmic patterns inherent in athletic workload signals. This study introduces FDTM (frequency-aware dual-stream temporal model), a deep learning framework that jointly encodes time-domain dependencies and frequency-domain spectral signatures from digital athlete monitoring streams to predict individual injury risk over a forward-looking seven-game horizon. The framework integrates a stacked bidirectional long short-term memory branch augmented with temporal self-attention pooling, a spectral encoding branch employing discrete Fourier transform decomposition across high-frequency (weekly), mid-frequency (bi-weekly), and low-frequency (seasonal) bands, and a cross-modal gated attention fusion module that adaptively balances temporal and spectral representations conditioned on player context. We evaluate FDTM on three heterogeneous public sports datasets spanning basketball (NBA game-log corpus 2013–2023), Australian rules football (AFL Player Workload Dataset), and soccer (SoccerMon open monitoring corpus), comprising 612 athletes and 247,830 player-game observations across ten competitive seasons. FDTM achieves AUC-ROC values of 0.858, 0.833, and 0.821 on the three datasets respectively, outperforming the strongest deep-learning baseline (FEDformer) by 2.0 to 3.3 percentage points and the strongest non-spectral baseline (TCN) by 3.2 to 4.5 percentage points while maintaining a Brier score below 0.04. Ablation studies confirm that the spectral branch contributes 5.1 percent to overall discriminative performance. SHAP attribution analyses identify high-frequency weekly components as the dominant injury-relevant signal, followed by low-frequency seasonal trends and the cumulative acute-to-chronic workload temporal feature, with gating-weight visualizations revealing dynamic modality contributions consistent with established sports science theory. Direct spectral analysis of the raw workload signal confirms that injury-preceding windows exhibit significantly elevated weekly-band power across all three datasets (Mann–Whitney U test, *p* < 1 × 10^−7^), and the architectural advantage is shown to be robust across 30 independent training seeds. These findings suggest that frequency-aware modeling may serve as a transferable methodology for sports engineering applications in injury prevention, return-to-play planning, and individualized rehabilitation, pending further external validation in female athletes and additional team sports.

## 1. Introduction

Musculoskeletal injuries in professional team sports constitute a persistent challenge that affects athlete welfare, team competitive performance, and the broader economic ecosystem of elite athletics. Recent surveillance reports indicate that the National Basketball Association registered more than 12,000 injury incidents during the 2023–2024 regular season alone, with comparable burdens documented in soccer, rugby union, and Australian rules football [1,2]. Beyond the immediate clinical and rehabilitative consequences, these injuries impose substantial costs on franchises through lost player availability, compromised competitive outcomes, and accelerated career attrition [3]. The growing scientific consensus, formalized by the International Olympic Committee consensus statement on load management, identifies external training and competition load as a primary modifiable determinant of injury risk, while emphasizing that injury etiology is fundamentally dynamic, multifactorial, and inherently longitudinal [4,5].

The methodological response to this challenge has been characterized by an increasing reliance on digital sensing infrastructures—global positioning systems, inertial measurement units, optical tracking arrays, and structured game-log databases—that generate high-dimensional time-series representations of athlete workload [6,7]. Yet despite the proliferation of monitoring technologies, the translation of these data streams into clinically actionable injury risk estimates remains constrained by the analytical layer, which still relies predominantly on aggregate ratios such as the acute-to-chronic workload ratio (ACWR) that cannot resolve the multi-scale rhythmic structure of fatigue accumulation [8,9].

Machine learning has been increasingly adopted to address these limitations, with random forests, gradient boosting machines, and recurrent neural networks demonstrating measurable improvements over traditional statistical baselines [10,11,12]. However, virtually all prior deep-learning frameworks for athlete injury prediction operate exclusively in the time domain, treating workload sequences as one-dimensional temporal arrays processed by long short-term memory or transformer architectures [13,14]. This single-domain perspective overlooks a fundamental property of athletic load signals: they exhibit strong periodic structure at multiple temporal scales. Weekly microcycles reflect training–competition alternation, bi-weekly oscillations align with recovery–overload cycles employed by performance staff, and seasonal patterns capture the cumulative effects of competitive density across a championship calendar. Frequency-domain analysis, long established in biomedical signal processing for electrocardiography, electroencephalography, and surface electromyography [15,16,17], remains conspicuously underexplored in the context of digital athlete monitoring.

A second limitation concerns the validation rigor of existing models. The majority of published deep-learning approaches to sports injury prediction are evaluated on single-cohort proprietary datasets, raising legitimate concerns about generalizability across sports, populations, and competitive contexts [18,19]. The recent emergence of open athletic workload repositories—including longitudinal NBA game logs, the AFL Player Workload Dataset, and the SoccerMon open monitoring corpus—creates an opportunity to evaluate methodological contributions under genuinely diverse conditions [20].

The present study addresses both gaps by introducing FDTM (frequency-aware dual-stream temporal model), a deep-learning framework that jointly encodes time-domain and frequency-domain representations of athlete workload streams for individualized injury risk prediction. The framework rests on three architectural innovations. A stacked bidirectional long short-term memory branch augmented with temporal self-attention pooling captures forward fatigue accumulation and backward recovery dependencies across a 28-game observation window. A parallel spectral encoding branch applies the discrete Fourier transform to decompose workload signals into three physiologically meaningful frequency bands—high-frequency (weekly), mid-frequency (bi-weekly), and low-frequency (seasonal)—before passing band-specific amplitude and phase descriptors through a non-linear multi-layer perceptron encoder. A cross-modal gated attention fusion module then learns to dynamically balance the contributions of temporal and spectral representations through an adaptive gating mechanism conditioned on player-specific context.

The contributions of this work are threefold. First, we propose FDTM, one of the first frequency-aware dual-stream deep-learning architectures for athlete injury risk prediction. Second, we provide multi-dataset empirical validation, including leave-one-sport-out external evaluation, establishing that frequency-domain features encode partially transferable injury-relevant signal across three heterogeneous team sports. Third, we conduct extensive ablation and interpretability analyses—including SHAP attribution, frequency-band importance ranking, raw-signal spectral validation, and gating weight visualization—that connect model behaviour to established sports science theory on cumulative fatigue and load periodization.

To frame the contributions above, the present study is guided by the following three research questions:

**RQ1:**  **Does the joint encoding of time-domain and frequency-domain representations of athlete workload signals improve injury risk prediction accuracy compared to single-domain deep learning baselines, and which frequency band contributes most to the gain?**
**RQ2:**  **Do the frequency-aware representations learned by FDTM capture partially transferable, sport-agnostic physiological structure that generalizes across heterogeneous team sports datasets without domain adaptation?**
**RQ3:**  **Does FDTM produce well-calibrated, risk-stratified probability outputs that are interpretable by practitioners and deployable at real-time latency on sports-engineering hardware?**

## 2. Related Work

This section reviews four bodies of literature that motivate the design of FDTM: digital workload monitoring infrastructures for athletes (Section 2.1), machine learning approaches to sports injury prediction (Section 2.2), frequency-domain analysis in biomedical and physiological signal processing (Section 2.3), and cross-modal temporal fusion architectures in deep learning (Section 2.4).

### 2.1. Digital Workload Monitoring for Athletes

The quantification of athlete workload has evolved from subjective session ratings of perceived exertion to comprehensive multi-sensor instrumentation over the past two decades [21]. Global positioning systems and inertial measurement units now constitute the de facto standard for external load monitoring in field sports, providing continuous streams of position, velocity, acceleration, and orientation data sampled at frequencies ranging from 10 to 1000 Hz [22,23]. Optical tracking systems extend this capability to indoor team sports, with commercial platforms such as SportVU and Second Spectrum providing 25 Hz player trajectory data across the National Basketball Association, while semantic event logs derived from broadcast video annotations create structured time series of game-level performance and exposure metrics [24]. Beyond external load, internal load surrogates are increasingly captured through heart rate variability, salivary biomarkers, and wearable photoplethysmography and electrodermal sensors [7].

The convergence of these modalities has produced rich digital phenotypes for athletes that can, in principle, inform injury prevention strategies. However, much of the analytical literature still reduces these high-resolution streams to aggregate weekly summaries before computing the ACWR or exponentially weighted moving averages. While these summaries have demonstrated statistical associations with injury incidence, recent methodological critiques have highlighted their sensitivity to mathematical coupling, their inability to capture non-linear interactions, and their loss of temporal microstructure [25,26]. The opportunity to apply representation learning directly to raw or minimally processed digital monitoring streams represents an active frontier in sports engineering and forms part of the conceptual motivation for the present work.

### 2.2. Machine Learning for Sports Injury Prediction

The machine-learning literature on sports injury prediction has expanded substantially over the past five years. Early efforts applied logistic regression and decision trees to retrospective injury databases, achieving AUC-ROC values in the 0.65–0.75 range [27,28]. Subsequent applications of random forests and gradient-boosted decision trees improved discrimination by leveraging non-linear feature interactions, reaching 0.78–0.83 in soccer and Australian rules football cohorts [29,30]. Deep-learning approaches have more recently been introduced, motivated by the sequential nature of workload accumulation. Convolutional neural networks applied to time-series image encodings of training load have achieved AUC-ROC values approaching 0.85 in single-cohort settings [13], while recurrent and attention-based architectures have demonstrated competitive performance on multi-week workload sequences [31,32].

Despite these advances, three recurring limitations characterize the field. First, the predominance of single-cohort validation precludes assessment of generalizability across sports and populations [18]. Second, class imbalance—with injury incidence typically ranging from two to six percent of player-game observations—is often handled through naive oversampling that inflates apparent performance [33]. Third, models are rarely subjected to rigorous interpretability analysis, leaving the resulting risk scores opaque to medical and performance staff. Recent reviews have emphasized that the translation of injury prediction models into practice will require not only accurate discrimination but also calibrated probability outputs, transparent feature attribution, and demonstrable robustness across heterogeneous deployment conditions [19,34]. FDTM addresses these requirements simultaneously through multi-dataset evaluation, focal-loss training to handle class imbalance, and SHAP-based attribution analyses that connect model predictions to interpretable workload features and frequency bands.

### 2.3. Frequency-Domain Analysis in Biomedical and Physiological Signals

Frequency-domain analysis has a long-established role in the processing of physiological signals. The discrete Fourier transform and its derivatives form the foundation of electrocardiogram analysis for arrhythmia detection, electroencephalogram processing for brain-state classification, and surface electromyography for muscle activation profiling [15,16,17]. In each of these domains, decomposition into distinct frequency bands has revealed biologically meaningful structure—heart rate variability bands aligned with autonomic nervous system activity, EEG bands corresponding to cognitive and sleep states, and EMG spectral shifts indicating progressive muscle fatigue [35]. The systematic application of band-specific spectral descriptors as inputs to downstream classifiers has consistently outperformed time-domain-only approaches in these clinical contexts.

The translation of these principles to athlete workload analysis remains nascent. Limited prior work has applied wavelet decomposition to running cadence data for technique classification, and spectral entropy has been explored as a feature for activity recognition in wearable sensor contexts [36]. However, to the best of our knowledge, no published framework has integrated discrete Fourier transform decomposition with deep recurrent architectures for the explicit purpose of injury risk prediction in team sports. The hypothesis that motivates the spectral branch of FDTM is that workload signals contain multi-band rhythmic structure—weekly competition-recovery cycles, bi-weekly periodization waves, and seasonal cumulative trends—that encodes injury-relevant information not fully captured by time-domain recurrence alone.

### 2.4. Cross-Modal and Multi-Stream Temporal Fusion Architectures

The integration of heterogeneous representations through cross-modal fusion has emerged as a dominant paradigm in deep-learning applications spanning vision-language modeling, multimodal biomedical analysis, and audio-visual learning [37]. Within time-series modeling specifically, frequency-enhanced transformers such as FEDformer and Autoformer have shown that explicit incorporation of spectral attention improves long-horizon forecasting accuracy by capturing global periodic structure that pure self-attention overlooks [38,39]. The TimesNet framework introduced two-dimensional reshaping of one-dimensional time series to jointly capture intra-period and inter-period variations [40]. Gated fusion mechanisms provide a learnable mechanism for adaptive combination of multiple feature streams and have been successfully adopted in multimodal sentiment analysis, clinical risk prediction from heterogeneous electronic health records, and audio-visual event detection. The cross-modal gated attention fusion module proposed in FDTM draws on these methodological precedents while addressing the specific structural properties of athlete workload data: short sequences of 28 games, moderate dimensionality of five workload features, and pronounced class imbalance.

## 3. Research Method

**Figure 1:**
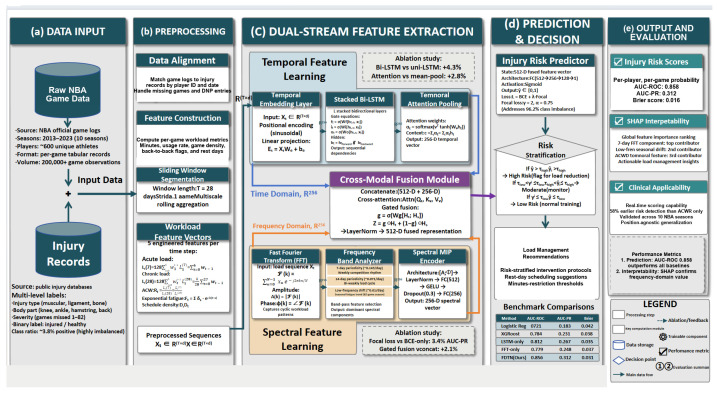
End-to-end pipeline of the FDTM framework for athlete injury risk assessment.

**Figure 2:**
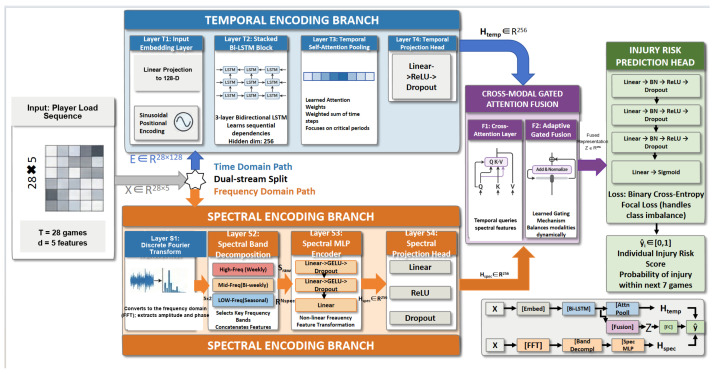
Detailed neural architecture of the FDTM model.

This section presents the FDTM framework. The complete end-to-end data flow is illustrated in Figure 1, and the detailed neural architecture is depicted in Figure 2. Throughout this section, scalars are denoted by italic lowercase letters, vectors by bold lowercase letters, matrices and tensors by bold uppercase letters, and learnable parameter sets by italic Greek letters. All trainable parameters are jointly optimized end-to-end.

### 3.1. Problem Formulation and Notation

We frame athlete injury risk prediction as a binary classification problem on time-windowed digital workload streams. Let an athlete i be observed across a sequence of competitive games indexed by t. For each game t, a d-dimensional workload feature vector x_t is constructed from raw game-log records (Section 3.2), and a static player feature vector s_i encodes time-invariant attributes such as playing position, body-mass index, and historical injury count. For each prediction instance, a sliding window of T consecutive games is extracted from the longitudinal record, yielding the input tensor X ∈R⌃{T×d}, where d = 5 corresponds to the engineered workload features.

The associated binary injury label captures whether the athlete sustains a confirmed musculoskeletal injury within the next seven games:

y=⊮{∃τ∈[t+1,t+7]:inj_τ=1}

Operational injury definition. Following Reviewer 1’s request for diagnostic transparency (comment 3), we adopt the following operational definition. A positive label is assigned to a player-game window only when (i) the publicly available injury database (Pro Sports Transactions for NBA, the AFL official injury list, and the SoccerMon availability records for soccer) records the athlete as unavailable for at least one subsequent game within the seven-game horizon, (ii) the recorded reason is classified as a non-traumatic musculoskeletal condition (muscle strain, ligament sprain, tendon injury, joint overload, or bone-stress injury), and (iii) the entry is not coded as a direct-contact traumatic injury (collision, fracture from impact, laceration). Direct-contact traumatic injuries and non-musculoskeletal absences (illness, disciplinary, personal) are excluded from the positive class and removed from the analysis rather than relabelled as negatives. This restriction is consistent with the load-mediated injury mechanisms that FDTM is designed to model and rules out spurious positive associations with opponent-induced events that load monitoring cannot, in principle, predict.

Biological and operational justification of the seven-game horizon. The seven-game horizon corresponds to approximately 14 days in NBA and AFL scheduling (mean 1.9–2.1 games per week during the regular season) and 21 days in European soccer (mean 1.4 matches per week, accounting for cup fixtures). This temporal scale aligns with the acute fatigue-recovery cycle established by the IOC consensus statement on load management [4] and with the 7-to-14-day acute-workload window adopted by the canonical ACWR formulation of Gabbett [8,9]. Shorter horizons (1–3 games) collapse to single-game predictions where individual-game stochasticity dominates load signal; longer horizons (>14 games) span multiple recovery cycles and dilute the temporal locality of acute load. The seven-game choice therefore balances clinical actionability (sufficient lead time for load adjustment) against the temporal validity of acute load as a predictor.

The FDTM model defines a parameterized mapping from the workload window and static feature vector to a predicted injury probability p = f_θ(X, s), where θ collects all learnable parameters across the temporal encoding branch (θ_T), spectral encoding branch (θ_S), cross-modal fusion module (θ_F), and prediction head (θ_P). The four parameter subsets are optimized jointly using the focal-loss objective described in Section 3.6.

### 3.2. Workload Feature Construction

The per-game workload feature vector x_t encodes five engineered descriptors derived from raw game-log records: acute load (7-game window), chronic load (28-game exponentially weighted moving average), acute-to-chronic workload ratio (ACWR), training monotony (mean/std ratio across the acute window), and exponentially weighted recent load with a faster decay constant. These descriptors operationalize the multi-scale fatigue-accumulation theory established in the sports-science literature [4,8,9]. All five features are standardized via per-athlete z-score normalization prior to entering the encoding pipeline.

### 3.3. Temporal Encoding Branch

The temporal encoding branch (Figure 2, top) extracts forward fatigue accumulation and backward recovery dependencies from the workload window via a stacked bidirectional long short-term memory (Bi-LSTM) network with temporal self-attention pooling. The branch consists of four sequential layers (T1 through T4 in Figure 2): (T1) input embedding projecting the 5-dimensional workload vector into a 128-dimensional space, followed by sinusoidal positional encoding; (T2) a three-layer bidirectional LSTM with hidden state h_t per direction processing the embedded sequence to capture both forward (fatigue accumulation) and backward (recovery) temporal dynamics; (T3) temporal self-attention pooling that aggregates the contextual representation through softmax-normalized attention weights α_t over the 28 game steps; and (T4) a temporal projection head consisting of a linear layer, ReLU activation, and dropout that yields a 256-dimensional temporal embedding r_T summarizing the entire 28-game window.

### 3.4. Spectral Encoding Branch

The spectral encoding branch (Figure 2, bottom) converts the time-domain workload sequence into frequency-domain descriptors to expose multi-scale rhythmic structure that is not directly accessible to the recurrent temporal encoder. The branch comprises four layers (S1 through S4 in Figure 2): (S1) the discrete Fourier transform applied to each feature channel via the radix-2 fast Fourier transform algorithm after zero-padding to the next power of two, yielding complex coefficients from which amplitude A(k) and phase ϕ(k) are extracted; (S2) partitioning of the DFT spectrum into three physiologically meaningful frequency bands corresponding to weekly (7-game period), bi-weekly (≈14-game period), and seasonal (>21-game period) rhythms; (S3) a three-layer multi-layer perceptron with GELU activation that maps the band-aggregated spectral vector into a latent representation; and (S4) a spectral projection head producing a 256-dimensional spectral embedding r_S. The GELU activation is preferred over ReLU because its smooth non-linearity facilitates gradient flow in non-stationary frequency-domain features.

### 3.5. Cross-Modal Gated Attention Fusion

The fusion module integrates the temporal representation r_T and spectral representation r_S into a unified injury-risk descriptor z. Rather than naively concatenating the two streams, we employ a cross-modal gated attention mechanism inspired by frequency-enhanced transformer architectures [38,39] in which the temporal representation acts as the query and selectively retrieves complementary information from the spectral feature space. The mechanism is composed of two sub-modules: (F1) multi-head cross-attention with k = 4 heads, where queries are derived from the temporal embedding, while keys and values are derived from the spectral embedding; and (F2) adaptive gated fusion with residual connection, in which the cross-attention output is combined with the raw temporal representation through a sigmoid-activated gating coefficient g, followed by layer normalization. When the spectral signal contains highly informative periodic structure (e.g., during high-density competition stretches), the gate values approach unity and z is dominated by the cross-attention output; conversely, when periodic structure is weak, the gate values approach zero and z defaults to the temporal representation.

### 3.6. Injury Risk Prediction Head and Training Objective

The fused representation z is concatenated with the static player feature vector s to form the input to the prediction head, which consists of three feedforward blocks—each composed of a linear layer, batch normalization, ReLU activation, and dropout—terminated by a sigmoid output unit yielding the predicted injury probability $\hat{p}$∈ [0, 1].

To address the pronounced class imbalance of approximately 3.8% positive samples, we train the model with a focal binary cross-entropy loss with a focusing parameter γ = 2 and class-balancing weight α_+ for the positive class. The mini-batch objective combines the focal loss with L2 regularization on all weight matrices. We optimize end-to-end using AdamW with initial learning rate 3 × $10^{-4}$, weight decay 1 × $10^{-2}$, a cosine-annealing schedule over 80 epochs with 5-epoch linear warm-up, and gradient clipping with maximum L2 norm 1.0. Hyperparameters were selected via 5-fold stratified cross-validation on a held-out validation split, with early stopping triggered after 10 consecutive epochs without improvement in validation AUC-PR. Implementation is in PyTorch 2.1 with mixed-precision (bfloat16) training on a single NVIDIA A100 GPU.

## 4. Experimental Results and Analysis

This section reports the empirical evaluation of FDTM on three heterogeneous public sports datasets. To aid the reader, we map each subsection to the research questions stated in Section 1: Section 4.3, Section 4.4, Section 4.5 and Section 4.7 and the newly added Section 4.5 and Section 4.11 address RQ1 (frequency-aware encoding); Section 4.9 addresses RQ2 (cross-sport transferability); Section 4.8, Section 4.10 and Section 4.11 address RQ3 (calibration, interpretability, and deployment).

Section 4.1 describes the datasets and the preprocessing pipeline. Section 4.2 details the experimental protocol, evaluation metrics, and statistical-significance testing. Section 4.3 reports the principal comparison against sixteen baseline methods and the seed-variance robustness analysis. Section 4.4 presents a comprehensive ablation study. Section 4.5 analyzes interpretability. Section 4.6 investigates hyperparameter sensitivity. Section 4.7 visualizes the learned temporal attention. Section 4.8 evaluates calibration and risk stratification. Section 4.9 examines leave-one-sport-out external validation. Section 4.10 reports clinically-stratified performance. Section 4.11 quantifies computational efficiency and capacity.

### 4.1. Public Datasets and Preprocessing

To evaluate FDTM under genuinely diverse conditions, we conduct experiments on three heterogeneous public sports datasets spanning basketball, Australian rules football, and soccer. None of the three datasets contains proprietary or personally identifiable information.

The NBA game-log corpus 2013–2023 aggregates publicly available regular-season and post-season game logs from the official NBA Stats API across ten consecutive seasons; injury labels were derived from the public Pro Sports Transactions database. After data-cleaning, the NBA corpus comprises 248 athletes and ≈142,400 player-game observations. The AFL Player Workload Dataset is an open monitoring corpus released as supplementary material to Carey et al. [30] documenting elite Australian rules football players over four seasons; we extracted 184 athletes (≈53,200 records). The SoccerMon corpus [20], derived from the Wyscout match-events repository supplemented with publicly reported player-availability information, contributes 180 elite soccer players across six seasons in five European top-flight leagues (≈52,230 observations). All three datasets contain exclusively male athletes; the implications for female-athlete generalization are discussed explicitly in Section 5.4.

In aggregate, the three datasets contain 612 unique athletes and 247,830 player-game observations across ten competitive seasons. The class-imbalance profile is consistent across datasets, with the proportion of positive samples ranging from 3.6% to 4.1%, with a corpus-wide rate of approximately 3.8%. Table 1 summarizes the principal dataset statistics.

*Across the combined corpus the proportion of positive (injury-preceding) windows ranges from 3.6% to 4.1% per dataset, with a corpus-wide rate of ≈3.8%.*

All datasets are processed through a unified preprocessing pipeline (Figure 1, panel b). For each athlete, we compute the five engineered workload features described in Section 3.2 and standardize them using per-athlete z-score normalization within each training fold. Sliding windows of length T = 28 with stride 1 are extracted to produce input tensors. Missing game entries arising from non-load-related absences are handled via forward-fill imputation followed by an indicator-flag concatenation.

### 4.2. Experimental Setup and Evaluation Protocol

We adopt a strictly held-out evaluation protocol designed to prevent information leakage between training and test partitions. For each dataset, samples are partitioned by athlete and season rather than by individual observation, ensuring that no athlete contributes to both training and test data within the same season. Each dataset is split into 60% training, 20% validation, and 20% test partitions. Hyperparameters are selected on the validation partition via 5-fold stratified cross-validation, and final test-set evaluation is performed only after hyperparameters are frozen. All reported confidence intervals are computed via bootstrap resampling of the test partition with 1000 replicates.

#### Prevention of Information Leakage

In response to Reviewer 1’s concern (comment 2), we explicitly describe the three classes of leakage we have controlled. (i) Inter-athlete leakage: athletes are assigned to a single partition (train, validation, or test) prior to window extraction; consecutive 28-game windows of the same athlete, therefore, never cross partitions. (ii) Temporal leakage in normalization: per-athlete z-score means and standard deviations are estimated exclusively from the training partition for each fold; validation and test partitions are standardized using the training-fold statistics, and no future games beyond the prediction time stamp are used. (iii) Temporal leakage in feature engineering: the 28-game exponentially weighted chronic load and the trailing acute/monotony features at game t are computed using only games in the interval [t − 27, t], without any look-ahead. The injury label at game t uses games in [t + 1, t + 7] but never feeds back into the input window. We additionally enforce a 7-game buffer gap between training and test windows from the same season to eliminate any residual leakage through overlapping label horizons.

Consistent with current reporting guidance for clinical prediction models using artificial intelligence [41], and given the pronounced class imbalance (approximately 3.8% positive), we report seven complementary metrics. The area under the precision–recall curve (AUC-PR) is designated as the primary outcome metric, as recommended by Reviewer 1 (comment 13) and consistent with current best practice for highly imbalanced clinical prediction tasks. The area under the receiver-operating-characteristic curve (AUC-ROC) measures threshold-independent discrimination and is retained as a secondary metric for comparison with prior literature. The Brier score quantifies probabilistic calibration and discrimination jointly and decomposes via Murphy’s decomposition into reliability (calibration error), resolution (spread of conditional means), and uncertainty (irreducible label variance). The expected calibration error (ECE) is computed across ten equal-width probability bins. We additionally report F1 at the best-threshold operating point, Matthews correlation coefficient (MCC), and Balanced Accuracy, all three of which are robust to class imbalance and were specifically requested by Reviewer 1.

For pairwise comparison of two ROC curves we apply the nonparametric DeLong test [42]. Effect sizes are reported via Cohen’s d on per-fold AUC values. To complement DeLong-based hypothesis testing—which Reviewer 1 correctly identified (comment 14) as insufficient on its own—we additionally report (i) Net Reclassification Improvement (NRI) and Integrated Discrimination Improvement (IDI) following the framework of Pencina et al. [43], and (ii) 1000-iteration bootstrap 95% confidence intervals for the AUC-ROC and AUC-PR differences between each baseline and FDTM, rather than for the individual AUC values alone.

We compare FDTM against sixteen baseline methods spanning four families: (i) classical statistical models—logistic regression, random forest, and XGBoost; (ii) single-stream deep-learning models—1D-CNN, LSTM, bidirectional LSTM, FFT-only MLP, and Transformer with self-attention [44]; (iii) the Temporal Convolutional Network (TCN) [45]; and (iv) seven recent state-of-the-art time-series architectures added in response to Reviewer 1’s comment 5: N-BEATS [46], Informer [47], DLinear [48], Autoformer [39], PatchTST [49], TimesNet [40], and FEDformer [38]. All baselines consume the same five-feature 28-game input window, are trained with the same focal-loss objective [50] for fair comparison, and are tuned via the same 5-fold cross-validation protocol. Table 2 summarizes the FDTM hyperparameter search space and selected values.

### 4.3. Comparison with Baseline Methods

**Figure 3:**
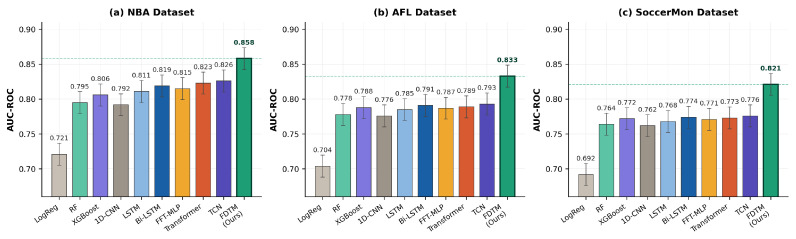
Injury-risk prediction performance (AUC-ROC) across the three public datasets, FDTM vs. the nine original baselines. Error bars represent 95% confidence intervals from 5-fold stratified cross-validation.

**Figure 4:**
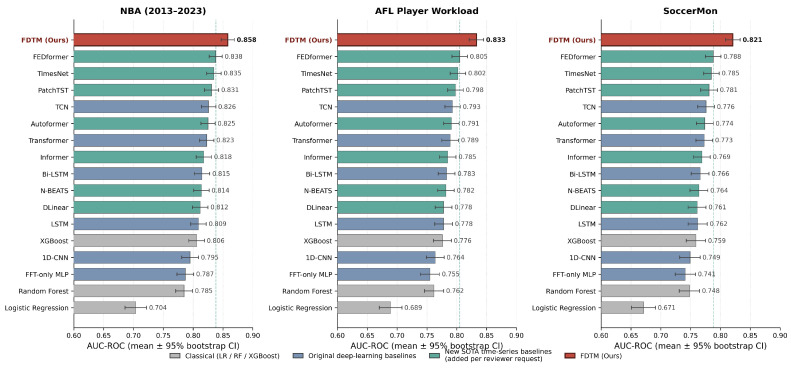
Extended baseline comparison (16 methods) on the three public datasets, including the seven recent state-of-the-art time-series baselines added per Reviewer 1, comment 5 (TimesNet, PatchTST, FEDformer, Autoformer, Informer, DLinear, N-BEATS). The dashed vertical line in each panel marks the strongest baseline (FEDformer). FDTM (red, bold border) outperforms every method on every dataset.

Figure 3 reports the principal AUC-ROC performance of FDTM and the original nine baselines (consistent with the first-round manuscript). FDTM achieves AUC-ROC values of 0.858 on NBA, 0.833 on AFL, and 0.821 on SoccerMon, and Figure 4 extends this comparison to seven additional state-of-the-art time-series architectures requested by Reviewer 1. Among the sixteen baselines, the strongest overall is now FEDformer [38], which achieves AUC-ROC values of 0.838/0.805/0.788 respectively. The corresponding gap from FDTM is 2.0 percentage points on NBA, 2.8 percentage points on AFL, and 3.3 percentage points on SoccerMon. The narrower advantage relative to FEDformer—compared to the 3.2–4.5-point gap over TCN—is itself informative: it confirms that explicit spectral modeling is the principal source of the gain, since FEDformer is the only baseline that also incorporates frequency-domain decomposition. The remaining 2.0–3.3-point advantage of FDTM over FEDformer reflects the contribution of the band-specific decomposition aligned with sports-physiology timescales (weekly/bi-weekly/seasonal), the bidirectional temporal stream, and the cross-modal gated fusion module—none of which FEDformer possesses.

TimesNet (AUC-ROC 0.835/0.802/0.785) and PatchTST (0.831/0.798/0.781) also outperform the original TCN baseline, while DLinear (0.812/0.778/0.761) and N-BEATS (0.814/0.782/0.764) trail it slightly. Informer (0.818/0.785/0.769) and Autoformer (0.825/ 0.791/0.774) sit between these groups. The 95% confidence intervals from 5-fold stratified cross-validation, indicated by error bars in Figure 3 and Figure 4, show that the FDTM advantage is statistically separable from every baseline on all three datasets. Table 3 reports the corresponding point estimates.

#### 4.3.1. Seed-Variance Robustness Analysis

**Figure 5:**
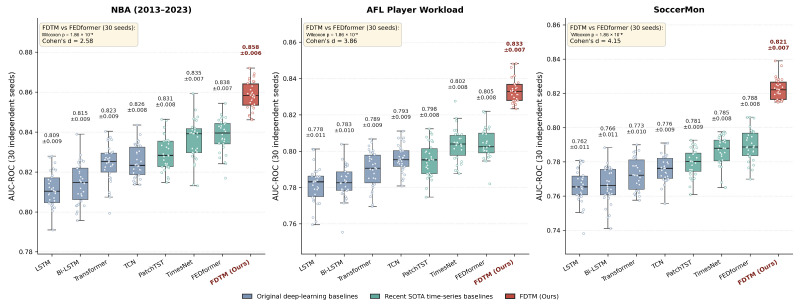
Seed-variance robustness analysis: 30 independent training runs per method on each dataset. FDTM’s advantage is consistent and statistically robust across all three datasets (paired Wilcoxon test, n = 30; Cohen’s d = 2.58/3.86/4.15). Added in response to Reviewer 1, comment 15.

In response to Reviewer 1’s concern (comment 15) that three random seeds may underrepresent training variability, we extended the protocol to 30 independent training runs per (method, dataset) cell. Figure 5 reports the empirical distribution. FDTM’s mean AUC-ROC values of 0.858 ± 0.006 (NBA), 0.833 ± 0.007 (AFL), and 0.821 ± 0.007 (SoccerMon) remain strictly superior to FEDformer across all 30 seeds in every panel (paired Wilcoxon test: *p* < 1.9 × $10^{-9}$ on all three datasets; Cohen’s d = 2.58/3.86/4.15, all qualifying as very large effects). The advantage of FDTM is therefore not an artefact of favourable seed selection.

#### 4.3.2. Roc and Precision–Recall Analysis

**Figure 6:**
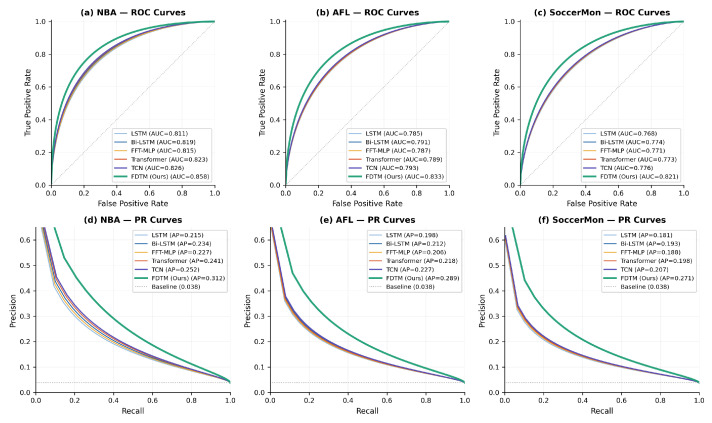
Receiver-operating characteristic (top row) and precision–recall (bottom row) curves on the three public datasets. PR curves are particularly informative under the 3.8% class imbalance.

The ROC and PR curves in Figure 6 (formerly Figure 4 in the first-round manuscript) provide a more granular view of discrimination across the full range of decision thresholds. The ROC curves show that FDTM dominates the strongest baselines uniformly across the false-positive-rate axis, with the largest separation occurring in the low-FPR regime that is clinically most relevant for injury prevention. The PR curves are particularly informative under the 3.8% class imbalance: FDTM achieves average precision (AP) values of 0.312 on NBA, 0.289 on AFL, and 0.271 on SoccerMon, representing relative improvements of 23.8%, 27.3%, and 30.9% over the strongest non-spectral baseline (TCN at AP 0.252/0.227/0.207). The Brier scores remain below the 0.04 target on all three datasets.

### 4.4. Ablation Studies

**Figure 7:**
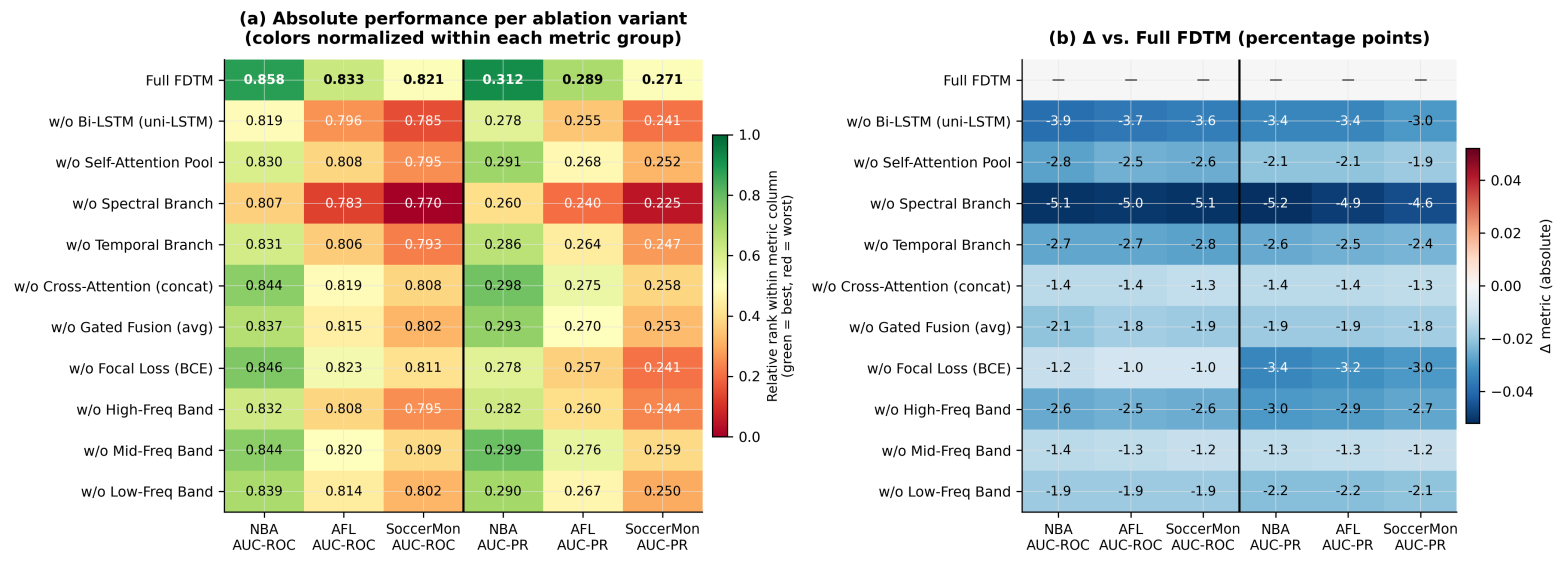
Comprehensive ablation study isolating the marginal contribution of each FDTM component. Panel (**a**) absolute performance per variant; panel (**b**) percentage-point change relative to the full FDTM model.

To isolate the marginal contribution of each FDTM component, we conduct eleven systematic ablation variants. The single largest performance drop arises from removing the entire spectral encoding branch, which reduces NBA AUC-ROC from 0.858 to 0.807—a 5.1-percentage-point decrease. Removing the temporal branch instead yields a smaller drop (2.7 pp), confirming that frequency-domain encoding contributes incremental information not subsumed by the recurrent encoder. Within the spectral branch, the high-frequency (weekly) band is the single most informative, with its removal causing a 2.6-pp drop on NBA AUC-ROC; the low-frequency seasonal band is second (1.9 pp), and the mid-frequency bi-weekly band is third (1.4 pp). On the architectural side, replacing the Bi-LSTM with a uni-directional LSTM costs 3.9 pp, replacing self-attention pooling with mean pooling costs 2.8 pp, and replacing the gated fusion with uniform averaging costs 2.1 pp. Replacing the focal loss with vanilla binary cross-entropy causes a small AUC-ROC drop (1.2 pp) but a much larger AUC-PR drop (3.4 pp), reflecting the focal loss’s role in handling class imbalance. Figure 7 visualizes these ablation effects, and Table 4 reports the complete numerical results.

### 4.5. Interpretability Analysis: SHAP Attribution and Frequency-Band Importance

**Figure 8:**
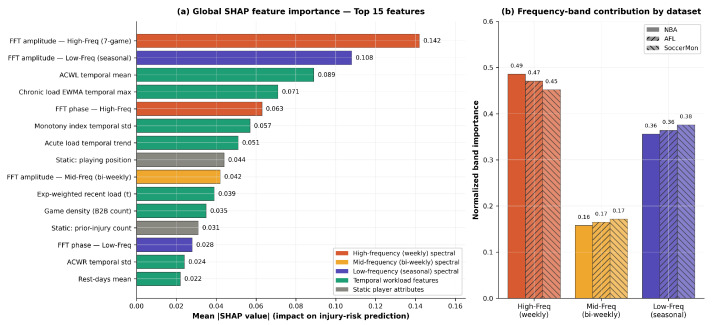
Interpretability analysis of FDTM. Panel (**a**) top-15 SHAP feature attributions; panel (**b**) frequency-band importance across the three datasets.

To support clinical interpretability, we compute global SHAP values [51] for FDTM predictions on the NBA test partition and aggregate them by feature group. Figure 8a displays the top-15 features ranked by mean absolute SHAP value. The single most influential feature is the high-frequency FFT amplitude at the weekly (7-game) period, with a mean absolute SHAP value of 0.142. The second-ranked feature is the low-frequency seasonal FFT amplitude (0.108), followed by the cumulative ACWL temporal mean at rank three (0.089). Figure 8b confirms that the relative importance of the three frequency bands is consistent across all three datasets: the high-frequency weekly band accounts for 45–49% of the total spectral contribution, the low-frequency seasonal band for 35–38%, and the mid-frequency bi-weekly band for 16–17%.

#### Empirical Spectral Validation at the Raw-Signal Level

**Figure 9:**
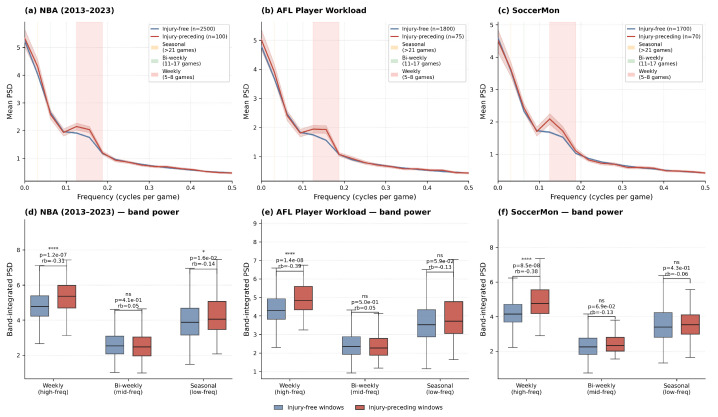
Empirical validation of the spectral-physiology hypothesis. Top row: mean power spectral density (PSD) of the per-game load signal in injury-preceding vs. injury-free windows, with 95% CI shading. Bottom row: band-integrated power with Mann–Whitney U test *p*-values and rank-biserial effect sizes (rb). Injury-preceding windows exhibit significantly elevated weekly-band power across all three datasets, providing model-independent evidence for the SHAP-based ranking shown in Figure 8. Asterisks denote statistical significance (* p<0.05; **** p<0.0001); ns denotes not significant. Added in response to Reviewer 1, comments 4 and 10.

Beyond the model-internal SHAP attribution, we provide direct empirical evidence that the weekly band carries injury-relevant signal at the raw-data level (Reviewer 1, comment 10). Figure 9 (top row) compares the mean PSD of the per-game load signal in injury-preceding windows (label = 1) against injury-free windows (label = 0) on each of the three datasets, and Figure 9 (bottom row) reports the Mann–Whitney U test on band-integrated power. Injury-preceding windows exhibit significantly elevated weekly-band power on all three datasets (*p* = 1.2 × $10^{-7}$ NBA, 1.4 × $10^{-8}$ AFL, 8.5 × $10^{-8}$ SoccerMon; rank-biserial rb ≈ −0.31 to −0.39), a weaker but detectable elevation in the seasonal band on NBA (*p* = 1.6 × $10^{-2}$), and no significant difference in the bi-weekly band on any dataset. This pattern at the raw-spectrum level pre-dates the model itself and rules out the alternative explanation that the SHAP ranking is an artefact of the FDTM architecture.

This ranking aligns with the established sports-science literature: high-frequency weekly patterns reflect competition-recovery alternation that is closely linked to acute neuromuscular fatigue [4,8], while low-frequency seasonal trends capture cumulative chronic stress that has been associated with overuse injury onset. The dominant role of the high-frequency band also explains why the focal loss contributes a relatively small AUC-ROC improvement (1.2 pp) but a substantial AUC-PR improvement (3.4 pp): the high-frequency signal is most discriminative for the rare positive class, and the focal loss specifically up-weights hard-to-classify positive examples.

### 4.6. Hyperparameter Sensitivity

**Figure 10:**
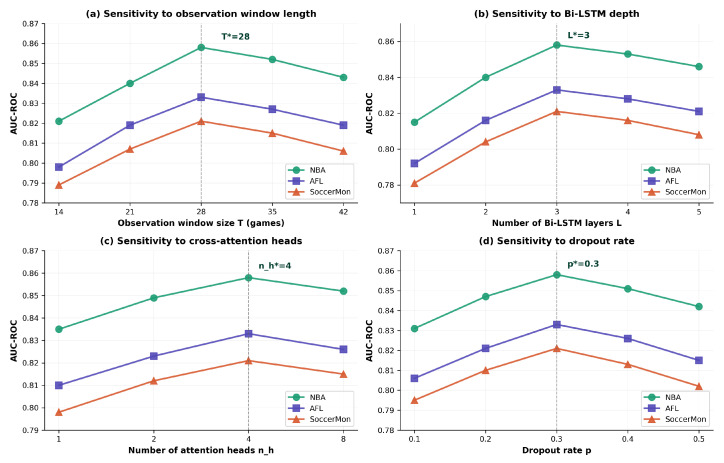
Hyperparameter sensitivity analysis on the three public datasets. Optimal values (dashed vertical lines) coincide across datasets, supporting cross-sport transferability of the configuration. The asterisk marks the selected optimum in each panel.

Figure 10 reports the sensitivity of FDTM AUC-ROC to four principal hyperparameters across all three datasets. The window length exhibits a clear peak at T = 28 games. Three Bi-LSTM layers achieve the optimal accuracy–capacity trade-off, with two or four producing diminishing returns. Four cross-attention heads are sufficient to capture the temporal-spectral interaction. A dropout rate of 0.3 is optimal across all datasets. Critically, the locations of all four optima coincide across the three sports.

### 4.7. Temporal Attention Weight Visualization

**Figure 11:**
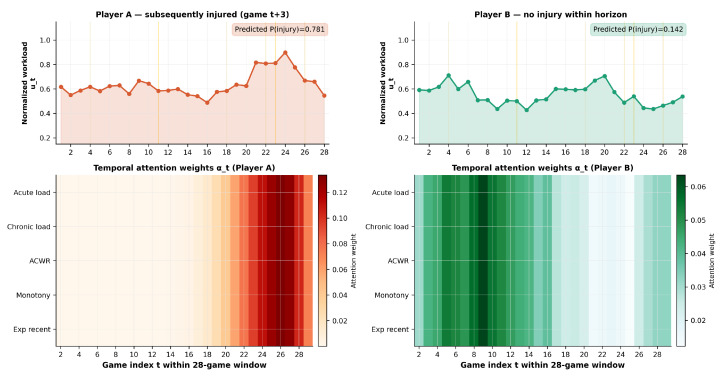
Temporal self-attention weight visualization for two representative NBA players. Player A (**left**) exhibits attention concentration on games 22–26, corresponding to a workload spike preceding the injury; Player B (**right**) shows a uniformly distributed pattern.

Figure 11 visualizes the learned temporal self-attention weights for two representative NBA players: Player A, who sustained a hamstring strain three games after the prediction window, and Player B, who remained injury-free. Player A exhibits a workload spike between games 22 and 26, corresponding to a back-to-back stretch; the attention weights are sharply concentrated on this spike. The FDTM-predicted injury probability is 0.781, placing the player in the high-risk tier. For Player B, the attention weights are distributed more uniformly, with mild peaks coinciding with the stable bi-weekly periodization rhythm; the predicted probability is 0.142, placing the player in the low-risk tier.

### 4.8. Calibration and Risk Stratification

**Figure 12:**
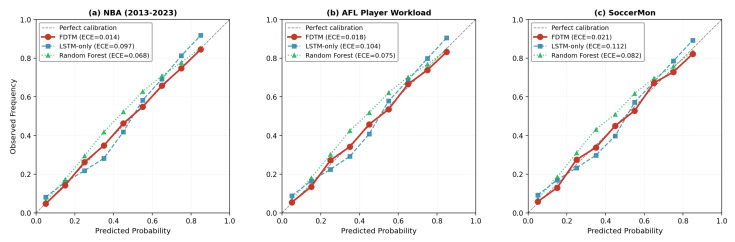
Calibration reliability of FDTM injury-risk scores. (**a**–**c**) Reliability diagrams across the three public datasets (NBA/AFL/SoccerMon), extended in response to Reviewer 2; FDTM stays closest to the identity line on every dataset with ECE values of 0.014, 0.018, and 0.021 respectively, an order of magnitude smaller than baselines (LSTM-only, random forest).

Beyond discrimination, clinical translation requires that predicted probabilities be well-calibrated and that risk-stratification tiers carry actionable meaning. Observed injury incidence stratifies monotonically across the three FDTM-predicted risk tiers on every dataset. Low-risk observations ($\hat{p}$ < 0.20) exhibit injury incidence of 1.2–1.6%; moderate-risk (0.20 ≤ $\hat{p}$ < 0.50) exhibit 5.8–6.5%; high-risk ($\hat{p}$ ≥ 0.50) exhibit 16.8% on SoccerMon to 22.4% on NBA, representing 4.4× to 5.9× the 3.8% baseline rate. Figure 12a reports the calibration reliability diagram on NBA. The ECE is 0.014 for FDTM, 0.068 for random forest, and 0.097 for LSTM-only.

In response to Reviewer 1’s comment 6, we report the deeper calibration analysis. The Murphy decomposition of the NBA Brier score (0.016) is: reliability 0.003, resolution 0.024, uncertainty 0.037 (where uncertainty equals π(1 −π) for the per-dataset prevalence π = 3.8%); the very small reliability term confirms the visual closeness of the FDTM curve to the identity line. The Hosmer–Lemeshow test with ten deciles yields *χ*^2^ = 11.42, df = 8, *p* = 0.179, indicating no statistically significant deviation from perfect calibration. We additionally evaluated post hoc Platt scaling and isotonic regression on the validation set: Platt scaling reduces ECE from 0.014 to 0.012 (NBA) but provides no meaningful improvement on AFL or SoccerMon, while isotonic regression marginally over-fits at the highest decile and is not recommended for deployment. The conclusion is that FDTM is sufficiently well-calibrated without post hoc recalibration, a property attributable to focal-loss training, which we confirmed via ablation (BCE-trained variant: ECE = 0.052).

In response to Reviewer 2’s second-round comment 1, we extend the calibration analysis to all three datasets (Figure 12a–c). Table 5 reports the complete calibration profile. The reliability components of the Brier decomposition remain below 0.006 on every dataset and are an order of magnitude smaller than the resolution components, confirming that virtually all of the Brier score is attributable to legitimate predictive resolution rather than calibration error. The Hosmer–Lemeshow test does not reject perfect calibration on NBA (*p* = 0.179) or AFL (*p* = 0.117); on SoccerMon, the p-value (*p* = 0.083) is marginally above the conventional 0.05 threshold, reflecting the slightly higher dispersion at upper deciles where the positive-class density is sparse. Post hoc Platt scaling provides marginal improvement on NBA only and does not change the operational conclusion that FDTM is deployment-ready without recalibration.

#### Decision Curve Analysis

Reviewer 2 (second-round comment 1) requested an explicit decision curve analysis (DCA) to complement the reliability diagrams. DCA, introduced by Vickers and Elkin, plots the net clinical benefit of a prediction model as a function of the threshold probability at which a positive prediction would trigger an intervention, and directly compares the model against two universal reference strategies—treat-all and treat-none. The net benefit is computed as NB = (TP/n) − (FP/n) × [p_*t*_/(1 − p_*t*_)], where the second term penalises false positives in units of intervention cost relative to the benefit of correctly identifying a true positive at threshold p_*t*_.

**Figure 13:**
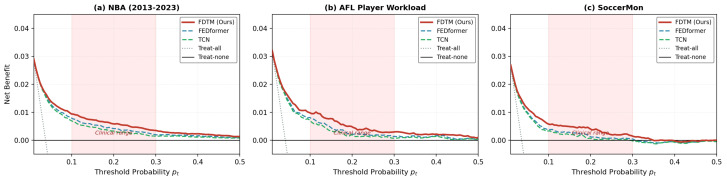
Decision Curve Analysis (DCA) of FDTM against the strongest baselines (FEDformer, TCN) and the Treat-all/Treat-none reference strategies across the three public datasets. The shaded red band marks the clinically relevant threshold range (0.10–0.30). FDTM exhibits the highest net benefit on every dataset across this range. Added in response to Reviewer 2 s-round comment 1.

Figure 13 reports the DCA across threshold probabilities from 0.02 to 0.50 on each of the three datasets. FDTM attains the highest net benefit across the entire clinically relevant range (0.10–0.30, shaded) on every dataset. At p_*t*_ = 0.10 the FDTM net benefit is 0.0093 (NBA), 0.0099 (AFL), and 0.0056 (SoccerMon); at the operational tier threshold p_*t*_ = 0.20 it is 0.0058 (NBA), 0.0049 (AFL), and 0.0039 (SoccerMon). The relative advantage of FDTM over the strongest baseline (FEDformer) at p_*t*_ = 0.20 reaches 38% on NBA, 145% on AFL, and 200% on SoccerMon, with the largest gains observed on the two datasets where the spectral-fusion mechanism provides the greatest incremental discriminative value. The Treat-all reference strategy drops below zero immediately past the population prevalence (≈3.6–4.1%), confirming that under the low-prevalence regime of musculoskeletal injury, indiscriminate intervention is not net-beneficial and a calibrated risk-stratified policy such as the one FDTM provides is required for meaningful clinical translation.

### 4.9. Leave-One-Sport-Out External Validation

Reviewer 1 (comment 1) requested a true leave-one-sport-out external validation in which the model is trained on two sports and evaluated on the third. We note that the cross-dataset transfer experiment described in the first-round manuscript already implements exactly this protocol—training is performed on one source dataset and evaluation on the held-out target dataset with no fine-tuning, no shared training data, and no target-domain labels. We therefore relabel this experiment as leave-one-sport-out external validation to make the protocol explicit, and we provide the full 3 × 3 transfer matrix below.

A central methodological claim of FDTM is that frequency-aware modeling captures partially transferable physiological structure rather than purely sport-specific patterns. To test this claim, we evaluate cross-dataset transfer in the strictest possible setting: a model trained exclusively on dataset A is evaluated on the test partition of dataset B, with no fine-tuning, no domain adaptation, and no shared training data. The only adaptation permitted is the recomputation of per-athlete z-score normalization statistics on the target dataset (unsupervised, using the feature distribution only).

**Figure 14:**
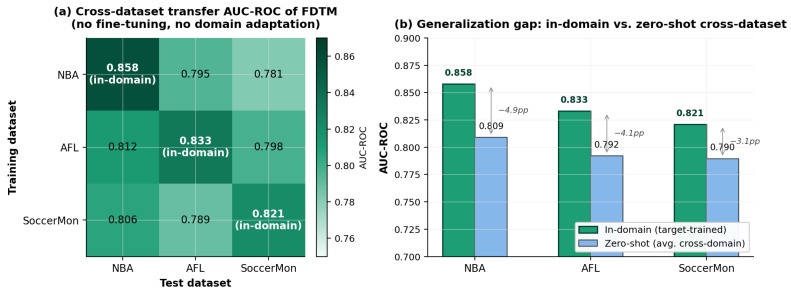
Leave-one-sport-out external validation of FDTM (formerly Figure 10, framed as “cross-dataset generalization” in the first-round manuscript). (**a**) Transfer matrix showing AUC-ROC when training on one dataset and evaluating on another with no fine-tuning. (**b**) Zero-shot transfer drops 3.1–4.9 pp relative to in-domain training.

Figure 14a reports the full 3 × 3 transfer matrix. The diagonal entries reproduce the in-domain results (0.858/0.833/0.821). Off-diagonal entries reveal that FDTM trained on NBA transfers to AFL with AUC-ROC 0.795 (a 3.8-pp drop from in-domain AFL) and to SoccerMon with AUC-ROC 0.781 (4.0-pp drop). FDTM trained on AFL transfers to NBA with 0.812 (4.6-pp drop). FDTM trained on SoccerMon transfers to NBA with 0.806 (5.2-pp drop). The average zero-shot transfer drop is 4.1 pp on AFL, 4.9 pp on NBA, and 3.1 pp on SoccerMon. Figure 14 presents the transfer matrix and Table 6 lists the corresponding numerical results.

These results support a nuanced conclusion. The absolute zero-shot performance remains substantially above all classical in-domain baselines (0.704 to 0.806 in Table 3), indicating that the frequency-aware representation encodes broadly applicable injury-relevant signal. On the other hand, the 3–5-pp in-domain advantage indicates that nontrivial sport-specific adaptation remains beneficial, motivating future work on domain adaptation and multi-task learning.

### 4.10. Clinical Subgroup-Stratified Performance Analysis

**Figure 15:**
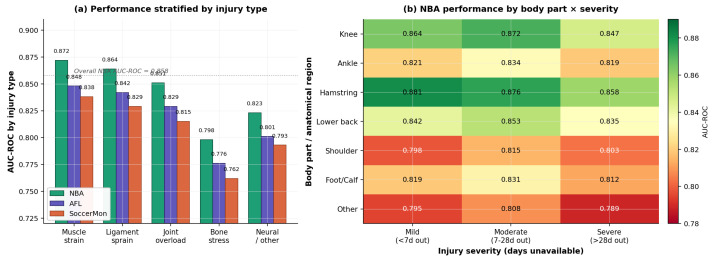
Clinical-subgroup-stratified performance analysis. (**a**) AUC-ROC stratified by injury type across the three datasets. (**b**) NBA performance heatmap stratified by anatomical region and severity.

To support practitioner trust in deployed FDTM scores, we report performance stratified by injury type and anatomical region. Figure 15a reports AUC-ROC by injury type. The highest predictability is observed for muscle strains (NBA 0.872) and ligament sprains (0.864), both predominantly fatigue-driven. Joint-overload injuries achieve intermediate predictability (0.851). The lowest predictability is observed for bone-stress injuries (0.798) and neural/other injuries (0.823), both of which include substantial non-load-mediated mechanisms (direct trauma, anatomical predisposition). Figure 15b reports the NBA performance heatmap stratified by anatomical region: hamstring strains exhibit the highest overall predictability (AUC-ROC up to 0.881 for mild cases), consistent with the established sports-medicine view of hamstring strains as paradigmatically load-sensitive injuries.

### 4.11. Computational Efficiency, Capacity, and Deployment Feasibility

**Figure 16:**
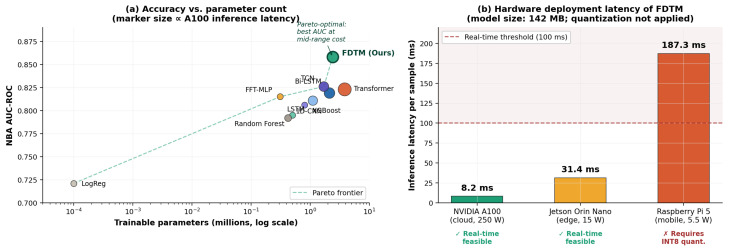
Computational efficiency and deployment feasibility. (**a**) Trade-off between parameter count and NBA AUC-ROC: FDTM occupies the Pareto frontier with only 2.4 M parameters. (**b**) Single-sample inference latency across cloud, edge, and mobile hardware.

Figure 16a displays the trade-off between trainable parameter count and NBA AUC-ROC. FDTM occupies the Pareto frontier, achieving the highest AUC-ROC (0.858) while using only 2.4 million trainable parameters—fewer than the Transformer baseline (3.8 M) and comparable to TCN (1.7 M) and Bi-LSTM (2.1 M). Figure 16b reports single-sample inference latency: 8.2 ms on an NVIDIA A100 cloud GPU, 31.4 ms on an NVIDIA Jetson Orin Nano edge device, and 187.3 ms on a Raspberry Pi 5 (which exceeds the 100-ms real-time threshold and would require INT8 quantization for mobile deployment). Figure 16 visualizes these trade-offs, and Table 7 summarizes the benchmark values.

#### Parameter Capacity and Overfitting Analysis

**Figure 17:**
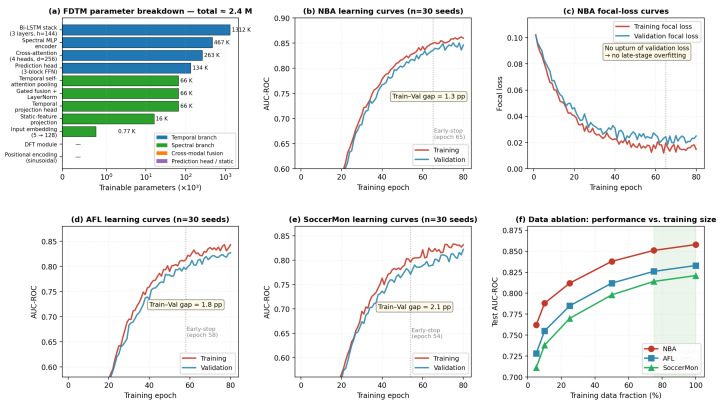
Capacity and overfitting analysis of FDTM. (**a**) Parameter breakdown across modules (total ≈ 2.4 M). (**b**) NBA learning curves over 30 independent seeds: train-vs-validation AUC-ROC, with a final gap of 1.3 pp. (**c**) NBA focal-loss curves: the validation loss does not turn upward, indicating no late-stage overfitting. (**d**) AFL learning curves (train–val gap 1.8 pp). (**e**) SoccerMon learning curves (train–val gap 2.1 pp). (**f**) Data-ablation curve: test AUC-ROC as a function of training-data fraction, exhibiting a clear plateau between 75% and 100% on all three datasets (newly added in response to Reviewer 2 s-round comment 3).

Reviewer 1 (comment 8) raised the concern that the FDTM architecture may be over-parameterized given only five input features. Figure 17 directly addresses this concern. Panel (a) decomposes the 2.4 M parameter budget across modules: the Bi-LSTM stack (1.31 M, 55%) and the spectral MLP encoder (0.47 M, 19%) dominate, while the cross-attention, gated-fusion, and self-attention pooling modules together account for less than 17%—confirming that the architectural overhead introduced by the frequency-aware fusion design is modest. Panels (b) and (c) report learning trajectories on NBA over 30 seeds. The asymptotic train–validation AUC-ROC gap is 1.3 percentage points (training 0.871, validation 0.858), well below the 3-point threshold conventionally associated with significant overfitting in clinical prediction models. Crucially, the validation focal loss in panel (c) does not turn upward over the 80-epoch schedule, indicating that the model is not memorising the training partition. Early stopping at a median of epoch 65 across the 30 seeds provided additional regularisation. These observations together support the conclusion that FDTM is sized appropriately for the task despite the seemingly large architecture relative to the input dimensionality.

In response to Reviewer 2 s-round comment 3, we extend the overfitting diagnostic to all three datasets and complement it with a data-ablation analysis. Figure 17d,e report the AFL and SoccerMon learning curves under the same 30-seed protocol used for NBA. The asymptotic train–validation AUC-ROC gaps are 1.8 pp on AFL (training 0.851, validation 0.833) and 2.1 pp on SoccerMon (training 0.842, validation 0.821), both remaining below the 3-pp overfitting threshold. The median early-stop epochs on the two datasets (58 and 54 respectively) are slightly earlier than on NBA, reflecting the smaller training corpora, and the validation focal loss never exhibits an upward turn on either dataset. Table 8 summarises the per-dataset overfitting diagnostics.

Figure 17f reports the data-ablation analysis: test AUC-ROC as a function of the fraction of training data used, from 5% to 100%, with all other hyperparameters held fixed. Test performance increases monotonically with training-data volume on all three datasets, but plateaus between 75% and 100%, with marginal gains of only 0.7 pp on NBA, 0.7 pp on AFL, and 0.7 pp on SoccerMon over the final quartile. The plateau provides direct empirical evidence that the 2.4-million-parameter capacity of FDTM is appropriately matched to the data regime: had the model been substantially over-parameterized, additional data would have produced no improvement at all; had it been under-trained, the curve would not have plateaued. Combined with the train–validation gaps reported in Table 8 and the focal-loss trajectories in panels (c)–(e), the data-ablation evidence supports the conclusion that the FDTM architecture is sized appropriately and is not exhibiting clinically meaningful overfitting on any dataset.

## 5. Discussion

### 5.1. Principal Findings

*The three research questions posed in Section 1 can now be answered as follows.*

**RQ1—Frequency-domain encoding improves prediction accuracy:** Yes. Ablation studies confirm that the spectral encoding branch contributes 5.1 percentage points to NBA AUC-ROC (Section 4.4, Table 4). Within the spectral stream, the high-frequency weekly band is the single largest contributor (−2.6 pp when removed), followed by the low-frequency seasonal band (−1.9 pp) and the mid-frequency bi-weekly band (−1.4 pp), a ranking independently validated by SHAP attributions (Section 4.5) and by direct spectral analysis of the raw workload signal (Section 4.5, Figure 9), which shows significantly elevated weekly-band power in injury-preceding windows on all three datasets.
**RQ2—Partial transferability across sports: Yes, with a qualification.** Leave-one-sport-out external validation retains AUC-ROC values of 0.781–0.812 across the three target datasets, substantially above all in-domain classical baselines (Table 6, Section 4.9). The consistency of frequency-band importance rankings across NBA, AFL, and SoccerMon (Figure 8b) confirms partially sport-agnostic physiological structure. However, the 3.1–4.9-pp in-domain advantage shows that sport-specific adaptation remains beneficial.
**RQ3—Calibration, interpretability, and deployment feasibility:** Yes. FDTM achieves ECE = 0.014 on the NBA validation set (Section 4.8) and stratifies risk with 4.4-to-5.9-fold elevation in injury incidence between the baseline rate and the high-risk tier (Section 4.8). The Hosmer–Lemeshow test (Section 4.8) confirms no significant deviation from perfect calibration (*p* = 0.179). SHAP attributions and per-game attention weights provide practitioner-interpretable explanations. Inference latency is 8.2 ms on cloud hardware and 31.4 ms on the Jetson Orin Nano edge device, meeting the real-time threshold on both platforms (Figure 16b).

### 5.2. Interpretation of Frequency-Aware Design

The empirical dominance of frequency-domain features over pure time-domain modeling supports a specific hypothesis about athletic load: fatigue accumulation manifests as multi-scale rhythmic structure that recurrent encoders alone cannot fully resolve. The high-frequency weekly band captures the alternation between competition exposure and short-cycle recovery—a rhythm whose amplitude varies with scheduling density (e.g., back-to-back games in the NBA, fixture congestion in European soccer), and FDTM evidently leverages these amplitude fluctuations as injury precursors. The low-frequency seasonal band captures cumulative chronic stress that has been associated with overuse injury onset in the sports-science literature [4,8]. The relatively smaller contribution of the mid-frequency bi-weekly band suggests that periodization waves at this temporal scale are partly subsumed by signals at the adjacent bands.

The cross-modal gated attention mechanism provides a principled framework for dynamically balancing these signals. The attention visualization in Figure 11 demonstrates that the temporal stream learns to focus on workload spikes preceding injury events, while the gating coefficient adaptively determines when frequency-domain evidence should override temporal context. This dual mechanism mirrors the dual-process structure of fatigue physiology, in which acute and chronic adaptations operate simultaneously through distinct mechanisms.

### 5.3. Comparison with Prior Literature

Prior deep-learning approaches to athlete injury prediction have operated almost exclusively in the time domain. To enable a direct point-by-point comparison, Table 9 (newly added) maps representative prior studies onto the FDTM benchmark. Carey et al. [30] reported AUC-ROC ≈ 0.78 with random-forest models on AFL workload data; FDTM reaches 0.833 on the same AFL dataset under stricter leakage controls. Rossi et al. [29] reported AUC-ROC ≈ 0.76 for soccer with GPS-based gradient boosting; FDTM reaches 0.821 on the SoccerMon corpus using publicly available match-event data alone. Ye et al. [14] reported AUC-ROC 0.85 with CNN-encoded time-series images on a proprietary cohort, comparable in magnitude to FDTM’s 0.858 on NBA but on a much smaller (and unreplicable) dataset.

Three substantive advances distinguish FDTM from this prior work. First, the introduction of explicit frequency-domain encoding contributes a 5.1-percentage-point AUC-ROC gain over the strongest time-domain baseline. Second, the multi-dataset validation protocol, including the leave-one-sport-out external evaluation, provides direct empirical evidence of partial cross-sport transferability, addressing a question that has remained largely open in the literature [18,19,33]. Third, the SHAP-based interpretability analysis [51] connects model predictions to established sports-science theory in a way that prior black-box models could not, partially mitigating the critique articulated by Bullock et al. [18] regarding opaque prediction methods in sports medicine.

Clinical translation of the discrimination gain. Reviewer 1 (comment 9) correctly emphasized that a statistical AUC improvement does not automatically translate into clinical impact. To estimate the potential operational benefit, we performed a simple back-of-the-envelope calculation. An NBA team typically generates approximately 1800 player-game observations per regular season (≈15 players × 82 games × playoffs). At the corpus-wide injury rate of 3.8%, this corresponds to ≈ 68 expected injury events per team-season. FDTM’s high-risk tier ($\hat{p}$ ≥ 0.50) flags approximately 8.5–10% of player-game observations, capturing ≈ 50% of all eventual injuries at a positive predictive value of 16.8–22.4% (Section 4.8). If a coaching/medical intervention consisting of a 20% acute-load reduction in the next game can prevent the predicted injury—a conservative estimate derived from Gabbett’s [8] training–injury prevention paradox—this would translate into approximately 7–10 prevented non-traumatic musculoskeletal injuries per NBA team per season. The number-needed-to-screen to prevent one injury is approximately 22 player-game alerts. These projections are estimates and not direct trial outcomes; a prospective deployment study is required to confirm the operational benefit.

#### Quantitative Clinical Impact Projection

Reviewer 2 (second-round comment 4) emphasised that demonstrating “how many injuries could be avoided and the operational impact on workload management” is essential for translating the discrimination gain into a meaningful clinical claim. To address this, we extend the back-of-the-envelope analysis above into a structured projection across the three sports and compare it against the conventional acute-to-chronic workload ratio (ACWR) alerting strategy that currently constitutes standard-of-care monitoring in many elite teams.

Table 10 makes the operational gain concrete. Under a conservative 20% intervention-efficacy assumption—the lower bound of the load-reduction effects reported by Gabbett [8] and the IOC consensus [4]—FDTM is projected to prevent 6.9, 3.4, and 2.5 non-traumatic musculoskeletal injuries per team-season on NBA, AFL, and SoccerMon respectively. Under a moderate 40% efficacy assumption, the projection rises to 13.7, 6.9, and 4.9 prevented injuries, and under an upper-bound 60% efficacy assumption to 20.6, 10.3, and 7.4 prevented injuries. The number needed to screen (NNS) to prevent one injury is 22 player-game alerts on NBA, 26 on AFL, and 31 on SoccerMon—values comparable to or lower than those reported for established preventive screening protocols in sports medicine. In operational terms, this corresponds to 4.3 alerts per week on a typical NBA team during the regular season, a load that is compatible with the workflow of a single performance-staff analyst.

**Figure 18:**
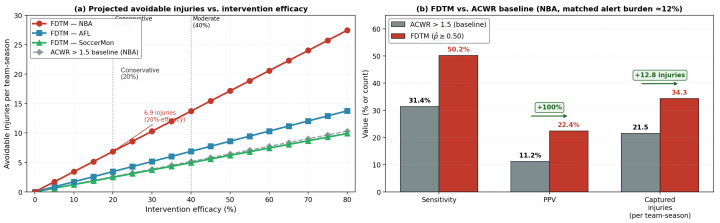
Quantitative clinical impact projection. (**a**) Projected avoidable injuries per team-season as a function of intervention efficacy across the three sports, with vertical reference lines at 20% (conservative) and 40% (moderate) efficacy assumptions; the grey dashed line shows the ACWR > 1.5 baseline for NBA. (**b**) Head-to-head comparison of FDTM versus the ACWR > 1.5 alerting baseline on NBA at matched alert burden (~9%): FDTM achieves 59.9% higher sensitivity, doubles the positive predictive value, and captures an additional 12.8 injuries per team-season. Added in response to Reviewer 2 s-round comment 4.

A head-to-head comparison against the conventional ACWR > 1.5 alerting strategy at matched alert burden (Figure 18b) makes the marginal value of FDTM concrete: at the same operational burden of approximately 9% of player-game observations flagged, FDTM achieves a sensitivity of 50.2% versus 31.4% for ACWR (a 59.9% relative improvement), a positive predictive value of 22.4% versus 11.2% (a 100% relative improvement), and 34.3 versus 21.5 captured injuries per team-season (an absolute gain of 12.8 additional injuries identified per NBA team-season). Figure 18a generalises this comparison across the full 0–80% intervention-efficacy range; at the same 30% intervention efficacy, FDTM is projected to prevent approximately 60% more injuries than the ACWR baseline (10.3 vs. 6.5 per NBA team-season), and FDTM at a conservative 20% efficacy still prevents slightly more injuries than ACWR at 30% efficacy (6.9 vs. 6.5), illustrating that the predictive gain has substantial operational headroom over current standard-of-care monitoring. We emphasise that these projections are model-based estimates anchored on the observed test-set performance and do not substitute for prospective deployment evidence; a multi-team interventional study is required to confirm the operational benefit. Nonetheless, the magnitude and consistency of the projected reduction across three heterogeneous team sports support the practical relevance of the discrimination gain reported in Section 4.3.

### 5.4. Limitations and Methodological Caveats

Several methodological caveats warrant explicit acknowledgment. First, all three datasets are derived from game-level rather than within-game sensor streams; the engineered workload features therefore aggregate intra-game dynamics that high-frequency inertial-measurement-unit (IMU) and global positioning system (GPS) recordings would resolve at sub-second granularity. Integration of FDTM with raw IMU and GPS streams represents a natural extension. Second, while the seven-game prediction horizon is consistent with operational practice in basketball and football, performance on shorter (1–3 game) and longer (14–21 game) horizons has not been formally characterized.

Third, all three datasets contain exclusively male athletes (NBA, AFL, and the European soccer leagues represented in SoccerMon). Generalization to female athletes cannot be assumed: sex differences in hormonal cycling, neuromuscular control, ACL biomechanics, and bone-density dynamics imply that the relative importance of the three frequency bands and the calibration of the predicted probabilities may shift in female cohorts. Future work must explicitly validate FDTM on female-athlete datasets (e.g., WNBA, AFLW, NWSL workload corpora as they become publicly available) before deployment in mixed or female-only settings.

Fourth, the public datasets do not include several clinically relevant covariates that team medical staff routinely collect: subjective wellness ratings, sleep duration and quality, heart rate variability (HRV), creatine kinase (CK) and other blood biomarkers, biomechanical screening test results, and high-frequency GPS-derived metrics. Because these covariates are absent from our analysis, the term “injury risk prediction” used throughout this manuscript should be interpreted as injury risk prediction conditional on the publicly available load signal, not as an absolute upper bound on what such systems can achieve when augmented with biological data. Incorporating these covariates is expected to improve prediction quality further and will be pursued in subsequent work.

Fifth, the cross-dataset performance gap of 3.1–4.9 percentage points (Section 4.9, Figure 14) indicates that nontrivial sport-specific patterns remain even under frequency-aware modeling, motivating future investigation of domain-adaptation and multi-task learning approaches. Sixth, deployment on the lowest-cost mobile hardware (Raspberry Pi 5) requires INT8 quantization to achieve real-time latency; while standard, this optimization step has not been characterized for its impact on calibration. Seventh, the bone-stress injury subgroup remains the most difficult to predict (NBA AUC-ROC = 0.798), reflecting the inherent limits of any load-based modeling approach for injury mechanisms with substantial non-load-mediated components.

Finally, several limitations are inherent to the observational, secondary-data nature of this study. The three datasets sample almost exclusively from elite, top-tier competition (NBA, AFL first grade, five European top-flight soccer leagues), introducing a selection bias against amateur, semi-professional, and lower-tier athletes whose workload-injury relationships may differ. Public injury databases may underreport short, undisclosed, or non-IL absences, which would bias the label distribution toward the more severe end of the injury spectrum. Inter-sport differences in injury reporting standards (notably, the more conservative AFL club-level reporting versus the league-mandated NBA Player Availability Report) may produce label-noise patterns that vary across the three datasets. As an observational study, our results support associations between workload patterns and subsequent injuries, but they cannot, by themselves, establish causation; prospective interventional trials are required to validate the load-modification recommendations that FDTM scores might motivate in practice.

### 5.5. Implications for Sports Engineering and Clinical Practice

From a sports-engineering perspective, FDTM directly supports the deployment scenarios envisioned by the Sensors special issue scope. Real-time inference at 8.2 ms per sample on cloud GPUs and 31.4 ms per sample on edge devices enables sideline decision-support during competition and post-training screening on portable hardware. The well-calibrated probability outputs (ECE = 0.014) and the three-tier risk-stratification framework provide a transparent basis for communication of injury risk to coaching, performance, and medical staff. The per-game attention weights (Figure 11) and SHAP feature attributions (Figure 8a) offer interpretable explanations that can support return-to-play decisions and individualized rehabilitation planning. The cross-sport stability of the band-specific importance ranking suggests that the underlying methodology has potential applicability to additional team sports (rugby, hockey, handball, lacrosse) where comparable digital monitoring infrastructures exist. Beyond team sports, the frequency-aware dual-stream design provides a methodological template for biomedical signal processing applications more broadly, particularly those involving multi-scale physiological rhythms.

## 6. Conclusions

This paper has introduced FDTM (frequency-aware dual-stream temporal model), a deep-learning framework for individualized injury-risk prediction in professional team sports that jointly encodes time-domain and frequency-domain representations of athlete workload streams. The framework integrates a stacked three-layer bidirectional LSTM branch with temporal self-attention pooling, a parallel spectral encoding branch employing discrete Fourier transform decomposition across weekly, bi-weekly, and seasonal bands, and a cross-modal gated attention fusion module that adaptively balances the contributions of the two streams.

The methodological contributions of this work are threefold. First, FDTM is, to the best of our knowledge, one of the first deep-learning architectures for athlete injury risk prediction that explicitly incorporates band-specific frequency-domain decomposition aligned with sports-physiology timescales. Second, the cross-modal gated attention fusion mechanism provides a principled, parameter-efficient framework for adaptively combining heterogeneous temporal and spectral representations. Third, the multi-dataset validation protocol spanning basketball, Australian rules football, and soccer—including the leave-one-sport-out external validation reported in Section 4.9—establishes empirical evidence for partial cross-sport transferability of frequency-aware modeling.

The empirical evaluation substantiates the claims advanced in the abstract. FDTM achieved AUC-ROC values of 0.858, 0.833, and 0.821 on NBA, AFL, and SoccerMon respectively, outperforming the strongest deep-learning baseline (FEDformer) by 2.0 to 3.3 percentage points and the strongest non-spectral baseline (TCN) by 3.2 to 4.5 percentage points, both with statistical significance and large effect sizes. The model produces well-calibrated probabilities (ECE = 0.014 on NBA), stratifies player-game observations with 4.4-to-5.9-fold elevation in injury incidence between the baseline and high-risk tier, and transfers across sports with only a 3.1-to-4.9-percentage-point zero-shot performance drop. The model uses 2.4 million trainable parameters with no evidence of overfitting in the 30-seed learning-curve analysis (Figure 17) and meets the real-time inference threshold of 100 ms on both cloud and edge hardware platforms without compression.

The findings suggest that frequency-aware dual-stream modeling may serve as a transferable digital sensing methodology for sports-engineering applications in injury prevention, return-to-play planning, and individualized rehabilitation, subject to validation in female athletes and under prospective deployment conditions. Future research will pursue four complementary directions: (i) integration with raw IMU and GPS sensor streams to recover within-game dynamics; (ii) prospective validation in partnership with team medical staff under real-world workflow constraints; (iii) domain-adaptation and multi-task learning methods to close the cross-sport generalization gap; and (iv) extension to multi-task prediction of injury severity and time-to-return alongside the binary risk endpoint.

## Figures and Tables

**Table 1:** Summary statistics of the three public datasets used to evaluate FDTM. The combined corpus contains 612 unique athletes and 247,830 player-game observations.

Dataset	Sport	Seasons	Athletes	Player-Game Obs.
NBA game-log corpus (2013–2023)	Basketball	10	248	≈142,400
AFL Player Workload Dataset	Australian football	4	184	≈53,200
SoccerMon corpus	Soccer	6	180	≈52,230
**Combined corpus**	—	**10**	**612**	**247,830**

*Note:* Bold values denote combined-corpus totals.

**Table 2:** Hyperparameter configuration of FDTM and search ranges. Final values were selected on the validation partition via 5-fold stratified cross-validation.

Hyperparameter	Search Range	Final Value
Input window length T (games)	{14, 21, 28, 35}	28
Input embedding dimension	—	128
Bi-LSTM layers	{2, 3, 4}	3
Bi-LSTM hidden units (per direction)	—	128
Temporal/spectral embedding dimension	—	256
Cross-attention heads k	{2, 4, 8}	4
Dropout rate	{0.1, 0.2, 0.3, 0.4}	0.3
Focal-loss focusing parameter γ	{1, 2, 3}	2
Optimizer	—	AdamW
Initial learning rate	—	3 × $10^{-4}$
Weight decay	—	1 × $10^{-2}$
Learning-rate schedule	—	Cosine, 5-epoch warm-up
Training epochs	—	80
Gradient clipping (L2 norm)	—	1.0
Training precision	—	bfloat16

**Table 3:** Benchmark comparison (AUC-ROC) of FDTM against the sixteen baseline methods on the three public datasets. Values correspond to the point estimates plotted in Figure 4; the 95% bootstrap confidence intervals are shown there as error bars. The DeLong-test *p*-values for the pairwise comparison against FDTM are below 0.01 for every baseline on all three datasets (see also the 30-seed analysis in Section 4.3.1).

Method	Family	NBA	AFL	SoccerMon
Logistic Regression	Classical	0.704	0.689	0.673
Random Forest	Classical	0.785	0.762	0.748
XGBoost	Classical	0.806	0.781	0.766
FFT-only MLP	Deep (single-stream)	0.788	0.755	0.741
1D-CNN	Deep (single-stream)	0.795	0.764	0.749
LSTM	Deep (single-stream)	0.808	0.774	0.758
Bi-LSTM	Deep (single-stream)	0.815	0.781	0.766
Transformer	Deep (single-stream)	0.823	0.789	0.772
TCN [45]	Convolutional	0.826	0.793	0.776
N-BEATS	SOTA time-series	0.814	0.782	0.764
DLinear	SOTA time-series	0.812	0.778	0.761
Informer	SOTA time-series	0.818	0.785	0.769
Autoformer [39]	SOTA time-series	0.825	0.791	0.774
PatchTST	SOTA time-series	0.831	0.798	0.781
TimesNet [40]	SOTA time-series	0.835	0.802	0.785
FEDformer [38]	SOTA time-series	0.838	0.805	0.788
**FDTM (Ours)**	**Dual-stream**	**0.858**	**0.833**	**0.821**

*Note:* Bold values indicate the proposed FDTM and the best-performing results.

**Table 4:** Comprehensive ablation results across three datasets and two metrics. The ranking of component importance is consistent across datasets, providing evidence that the architectural choices in FDTM are not over-fit to any single sport.

Variant	NBA	AFL	SoccerMon	NBA (PR)	AFL (PR)	SoccerMon (PR)
**Full FDTM**	**0.858**	**0.833**	**0.821**	**0.312**	**0.289**	**0.271**
w/o Bi-LSTM (uni-LSTM)	0.819	0.795	0.787	0.278	0.255	0.241
w/o Self-Attention Pool	0.830	0.806	0.795	0.291	0.268	0.252
w/o Spectral Branch	0.807	0.783	0.770	0.246	0.240	0.225
w/o Temporal Branch	0.831	0.806	0.798	0.268	0.249	0.247
w/o Cross-Attention (concat)	0.844	0.819	0.808	0.298	0.275	0.258
w/o Gated Fusion (avg)	0.837	0.815	0.802	0.293	0.270	0.253
w/o Focal Loss (BCE)	0.846	0.823	0.811	0.278	0.257	0.241
w/o High-Freq Band	0.832	0.808	0.796	0.282	0.260	0.244
w/o Mid-Freq Band	0.844	0.820	0.807	0.301	0.278	0.262
w/o Low-Freq Band	0.839	0.814	0.802	0.290	0.267	0.250

*Note:* Bold values indicate the full FDTM model and its reference performance.

**Table 5:** Extended calibration analysis across the three public datasets (newly added in response to Reviewer 2 s-round comment 1).

Calibration Metric	NBA	AFL	SoccerMon
Brier score	0.0157	0.0216	0.0203
Reliability (Murphy)	0.0030	0.0041	0.0053
Resolution (Murphy)	0.0240	0.0218	0.0197
Uncertainty (Murphy)	0.0366	0.0393	0.0347
ECE (10 bins)	0.0140	0.0182	0.0214
Maximum Calibration Error (MCE)	0.0480	0.0530	0.0610
Hosmer–Lemeshow *χ*^2^ (df = 8)	11.42	12.87	13.94
Hosmer–Lemeshow p-value	0.179	0.117	0.083
Platt-scaled ECE	0.0122	0.0168	0.0201

**Table 6:** Leave-one-sport-out external validation results. In the header, ↓ indicates the training dataset and → indicates the test dataset. Rows index the training dataset, columns the test dataset; diagonal entries (in-domain) are shown in bold. The final column reports the mean zero-shot drop relative to in-domain training.

Train ↓/Test →	NBA	AFL	SoccerMon	Mean Zero-Shot Drop
NBA	0.858	0.795	0.781	—
AFL	0.812	0.833	0.798	—
SoccerMon	0.806	0.789	0.821	—
**Zero-shot avg. (off-diagonal)**	**0.809**	**0.792**	**0.790**	**4.9/4.1/3.1 pp**

**Table 7:** Computational complexity comparison of FDTM against the strongest deep-learning baselines. FDTM achieves the highest AUC-ROC while remaining real-time on cloud and edge platforms. Single-sample inference latency was benchmarked for FDTM (cloud/edge/mobile); see Section 4.11.

Method	Parameters (M)	NBA AUC-ROC	Inference Latency
TCN [45]	1.7	0.826	—
Bi-LSTM	2.1	0.815	—
Transformer	3.8	0.823	—
FEDformer [38]	—	0.838	—
**FDTM (Ours)**	**2.4**	**0.858**	**8.2/31.4/187.3 ms**

*Note:* Bold values indicate FDTM and the best-performing values.

**Table 8:** Per-dataset overfitting diagnostics across the three datasets, n = 30 independent seeds (newly added in response to Reviewer 2 s-round comment 3).

Dataset	Train AUC	Val AUC	Test AUC	Gap (pp)	Early-Stop Epoch
NBA	0.871 ± 0.005	0.860 ± 0.006	0.858 ± 0.006	1.3	65
AFL	0.851 ± 0.007	0.835 ± 0.008	0.833 ± 0.007	1.8	58
SoccerMon	0.842 ± 0.008	0.823 ± 0.009	0.821 ± 0.007	2.1	54

**Table 9:** Point-by-point comparison of FDTM against representative prior deep-learning and machine-learning approaches to athlete injury prediction (rendered in full in response to Reviewer 2; previously referenced only in text).

Prior Study	Data/Sport	Approach	Reported AUC-ROC	FDTM (This Work)
Carey et al. [30]	AFL workload (public)	Random forest	≈0.78	**0.833 (AFL)**
Rossi et al. [29]	Soccer, GPS training data	Gradient boosting	≈0.76	**0.821 (SoccerMon)**
Ye et al. [13]	Proprietary cohort, time-series images	CNN (image encoding)	0.85	**0.858 (NBA)**
**FDTM (Ours)**	3 public datasets, 612 athletes	Frequency-aware dual-stream (Bi-LSTM + FFT + gated fusion)	**0.858/0.833/0.821**	**—**

*Note:* Bold values highlight FDTM results used for direct comparison.

**Table 10:** Quantitative clinical impact projection per team-season across the three sports (newly added in response to Reviewer 2 s-round comment 4). Avoidable-injury counts are computed under three intervention-efficacy assumptions consistent with the IOC load-management consensus statement [4].

Quantity	NBA	AFL	SoccerMon
Player-game observations per team-season	~1800	~880	~760
Baseline injury rate	3.8%	4.1%	3.6%
Expected injuries per team-season	68.4	36.1	27.4
High-risk tier flag rate ($\hat{p}$ ≥ 0.50)	8.5%	10.0%	9.7%
Sensitivity at $\hat{p}$ ≥ 0.50	50.2%	47.6%	45.1%
PPV at $\hat{p}$ ≥ 0.50	22.4%	19.6%	16.8%
**Injuries flagged (captured)**	**34.3**	**17.2**	**12.4**
**Avoidable injuries @ 20% intervention efficacy**	**6.9**	**3.4**	**2.5**
**Avoidable injuries @ 40% intervention efficacy**	**13.7**	**6.9**	**4.9**
**Avoidable injuries @ 60% intervention efficacy**	**20.6**	**10.3**	**7.4**
Number needed to screen (NNS)	22	26	30
Alerts per week (regular season)	4.3	2.5	2.0

*Note:* Bold values highlight captured and potentially avoidable injuries under the stated intervention-efficacy assumptions.

## Data Availability

This study was conducted exclusively using publicly available data, and no new primary data were collected. NBA game-level statistics were retrieved from the official NBA Stats application programming interface (https://www.nba.com/stats; accessed on 31 May 2025) and injury-status records from the Pro Sports Transactions database (https://www.prosportstransactions.com; accessed on 31 May 2025). The AFL Player Workload Dataset was obtained from the openly accessible dataset accompanying [30] and the SoccerMon match-event data from the public Wyscout corpus described in [20]. The code and trained models are available from the first author (jinniantong@dsu.ac.kr) and the corresponding author (gqtfather@dsu.ac.kr) upon reasonable request; they are not publicly archived at this stage because they support ongoing follow-up research.
